# Population pharmacokinetics and dose optimization of voriconazole in patients with COVID-19-associated pulmonary aspergillosis

**DOI:** 10.3389/fphar.2025.1554370

**Published:** 2025-04-09

**Authors:** Hui Wang, Yue Shen, Xuemei Luo, Lu Jin, Huaijun Zhu, Jing Wang

**Affiliations:** ^1^ Department of Infection Management, Nanjing Drum Tower Hospital, Nanjing, Jiangsu, China; ^2^ Department of Pharmacy, The First Affiliated Hospital of Xi’an Jiaotong University, Xi’an, Shaanxi, China; ^3^ Department of Pharmacy, Nanjing Drum Tower Hospital, Nanjing, Jiangsu, China

**Keywords:** COVID-19-associated pulmonary aspergillosis, voriconazole, population pharmacokinetics, dosage optimization, Monte Carlo

## Abstract

**Objectives:**

The study aimed to investigate the pharmacokinetic profile of voriconazole in patients with COVID-19-associated pulmonary aspergillosis (CAPA) to optimize dosing strategies.

**Methods:**

Population pharmacokinetic modeling was conducted using clinical data from CAPA patients to analyze voriconazole’s pharmacokinetic behavior. A one-compartment model with first-order elimination was employed to characterize voriconazole disposition. Covariate analysis was further utilized to evaluate the impact of continuous renal replacement therapy (CRRT) and select biochemical markers on voriconazole clearance.

**Results:**

The model estimated voriconazole’s apparent clearance (CL/F) at 3.17 L/h and apparent volume of distribution (V/F) at 135 L for a standard patient with CAPA. Covariates such as CRRT, C-reactive protein, gamma-glutamyl transpeptidase, aspartate aminotransferase, and platelet count were found to significantly influence voriconazole clearance. Monte Carlo simulations indicated that patients on CRRT required both a higher loading dose and an increased maintenance dose compared to those not on CRRT.

**Conclusion:**

This study provides an evidence-based guide for voriconazole dosing adjustments in CAPA patients, particularly for those undergoing CRRT. The findings emphasize the importance of individualized dosing to improve therapeutic outcomes in this high-risk population.

## 1 Introduction

Since the emergence of the COVID-19 pandemic, life-threatening secondary fungal infections have become a significant obstacle in the management of critically ill COVID-19 patients (Koehler et al., 2021a). COVID-19-associated pulmonary aspergillosis (CAPA) represents a common manifestation of invasive pulmonary aspergillosis (IPA) observed in intensive care unit (ICU) patients (Lescure et al., 2020; Feys et al., 2022; Hoenigl et al., 2022). The principal pathogenic fungi implicated in CAPA are *Aspergillus fumigatus* and *Aspergillus flavus*. Numerous cohort studies in the literature indicate an elevated risk of CAPA, with prevalence rates fluctuating between 2.5% and 47%, which correlates with a high mortality rate ranging from 22% to 74% in the ICU (Gioia et al., 2024; Hernández-Silva et al., 2024; Critical Care Medicine Group of Chinese Association of Chest Physicians and Chinese Thoracic Society, 2024; Bartoletti et al., 2021; Lamoth et al., 2021).

Global experts have recommended voriconazole (VRC) or isavuconazole as the first-line drugs recommended by European Confederation of Medical Mycology (ECMM) and the International Society for Human and Animal Mycology (ISHAM) for the treatment for possible, probable, or proven CAPA (Koehler et al., 2021b; Critical Care Medicine Group of Chinese Association of Chest Physicians and Chinese Thoracic Society, 2024). It is worth mentioning that voriconazole has been the preferred option in many Chinese hospitals, due to relatively easy to obtain and lower financial burden on patients (Han et al., 2023). Voriconazole, as a broad-spectrum second-generation triazole antifungal agent, is extensively utilized in the treatment of invasive fungal infections (IFIs) caused by pathogens such as *Aspergillus* and *Candida*. With a robust oral absorption profile boasting a 96% bioavailability, VRC provides clinical benefits whether given intravenously or orally (Greer, 2003). Nevertheless, it exhibits significant inter-individual variability and non-linear pharmacokinetics, largely due to saturable metabolic processes and genetic polymorphisms impacting CYP2C19, CYP2C9, and CYP3A4 enzymes. A large number of studies have indicated that the clinical efficacy and drug-related adverse reactions of VRC are closely related to its plasma concentrations. Subtherapeutic concentration may result in a higher probability of therapeutic failure. Whilst, surpassing its limited therapeutic range can lead to detrimental reactions such as hepatotoxicity, neurological disorders, and visual impairments (Mj and Aj, 2014). Generally, an adequate target exposure is recommended as a trough concentration of ≥1 or ≥2 mg/L to ensure sufficient effect, and kept at ≤5 mg/L to prevent VRC-related toxicity.

Critically ill patients, frequently characterized by an increased susceptibility to CAPA due to existing comorbidities, advanced age, dependence on invasive mechanical ventilation, renal replacement therapy (RRT), and administration of interleukin-6 inhibitors and corticosteroids, exhibit significant variability in their pharmacokinetics (Hoenigl et al., 2022; Gioia et al., 2024). As a result, for critically ill patients suffering from CAPA, who demonstrate a high mortality rate and significant pharmacokinetic variability, it is crucial to identify the determinants affecting the use of voriconazole in this population and to customize the therapeutic regimen accordingly. This strategy would consequently offer guidance for personalized and precise pharmacotherapy for these individuals.

The primary aim of this investigation was to assess the pharmacokinetic properties of voriconazole (VRC) in patients with CAPA employing nonlinear mixed effects modeling (NONMEM). Additionally, the study sought to quantify the degree of interindividual variability in VRC pharmacokinetics and to offer dosage recommendations derived from the finalized model to encourage the optimal utilization of VRC.

## 2 Materials and methods

### 2.1 Study design and ethics

The retrospective observational study entails the prospective accumulation of data for patients diagnosed with COVID-19 lung disease who were treated with voriconazole of Nanjing Drum Tower Hospital (Nanjing, China) from December 2022 to February 2023. The inclusion criteria of patients were as follows: 1) patients diagnosed with COVID-19 lung disease; 2) patients aged 18 years or above; 3) receiving an intravenous infusion of at least 72 h of voriconazole (Vfend^®^, 200 mg/bottle, Pfizer Pharmaceutical Co., Ltd.); and 4) blood sampling during the steady state for TDM was available. The exclusion criteria were: 1) incomplete clinical evaluation (unavailable data on renal function, biochemical indicators and other information); 2) pregnant and lactating women; 3) patients undergoing extracorporeal membrane oxygenation (ECMO); or 4) other factors deemed unsuitable for this study by the researchers. Study approval was granted by the Ethics Committee at Nanjing Drum Tower Hospital (No. 2023-338-01).

### 2.2 Data collection and definition

The study conducted an assessment of the variations in demographic characteristics among patients for model development. Clinical data were collected from a standardized format utilizing the hospital’s electronic medical record system, including sex (0 = female, 1 = male), age, weight (WT), alanine aminotransferase (ALT), aspartate aminotransferase (AST), alkaline phosphatase (ALP), glutamyl transpeptidase (GGT), total bilirubin (TBIL), direct bilirubin (DBIL), albumin (ALB), globulin (GLO), white blood cell count (WBC), neutrophil count (NEUT), hemoglobin (HGB), hematocrit (HCT), platelet (PLT), apolipoprotein A (APOA), apolipoprotein B (APOB), blood glucose (GLU), international normalized ratio (INR), urea, creatinine (CREA), C-reactive protein (CRP), creatinine clearance rate (CLCR), glomerular filtration rate (GFR), whether receiving CRRT (0 = no, 1 = yes) and co-medication of paxlovid, azvudine, clopidogrel, proton pump inhibitors (PPIs), calcium channel blockers (CCBs) and glucocorticoids. In addition, VRC dosing information was recorded, as well as blood samples for CYP2C19 genotypes (*2, *3, *17 alleles) detection and CYP3A4 genotype (rs4646437) were obtained from enrolled patients.

### 2.3 Quantification of voriconazole concentrations

At inclusion, patients received voriconazole at nearly 4 mg/kg twice a day with or without a loading dose of 6 mg/kg. Dosing interval, infusion rate and treatment course of voriconazole were determined by the treatment team. Voriconazole concentrations were assessed at a steady state following the administration of the fourth dose, and were subsequently analyzed utilizing High-Performance Liquid Chromatography (HPLC). This assay method was adapted from a previously published voriconazole assay method (Wang et al., 2024). A 300 uL sample of plasma was mixed with 600 uL of acetonitrile solution, which was vortexed for 30 s and centrifuged at room temperature for 8 min at 15,000×g. Following centrifugation, a 200 uL aliquot of the supernatant was injected into the analytical system. A mobile phase consisting of Acetonitrile: Milli-Q water (7:3 v/v) filtered through a 0.45um hydrophilic polypropylene filter was used for chromatographic separation on a C_18_ column (250 × 4.6 mm, 5 um) at a constant temperature of 40°C. The UV detector was set at 262 nm and the overall run time was 8 min. The calibration curves demonstrated acceptable linearity over 0.1–30 mg L^−1^ for voriconazole.

#### 2.3.1 CYP2C19 and CYP3A4 genotyping

Genomic DNA from whole blood was isolated using an Ezup Column Blood Genomic DNA Purification Kit (Sangon Biotech, Shanghai, China). Following quality control, the DNA fragments were carried out using the Sanger dideoxy DNA sequencing method by Sangon Biotech Co. (Shanghai, China) with automated ABI 3730XL DNA Analyzer (Applied Biosystems, Foster City, CA, USA).

SNPs involved in metabolic enzymes detected in the present study included CYP2C19*2 (rs4244285, c.681G>A), CYP2C19*3 (rs4986893, c.636G>A), CYP2C19*17 (rs12248560, c.-806C>T), and *CYP3A4* (rs4646437, c.671–202C>T). CYP2C19 phenotypes were classified into five categories: ultrarapid metabolizer (UM, CYP2C19*17/*17), rapid metabolizer (RM, CYP2C19*1/*17), extensive metabolizer (EM, CYP2C19*1/*1), intermediate metabolizer (IM, CYP2C19*1/*2, CYP2C19*1/*3, CYP2C19*2/*17, CYP2C19*3/*17) and poor metabolizer (PM, CYP2C19*2/*2, CYP2C19*2/*3, CYP2C19*3/*3). (Lee et al., 2022). The primers used are listed in Supplementary Table S1.

### 2.4 Population pharmacokinetic modeling

#### 2.4.1 Base model

Population PK models were developed utilizing NONMEM 7.3.0 (ICON Development Solutions, Ellicott City, MD, United States). Pirana (version 2.9.0) was employed to facilitate model development and data processing. The base model consists of structural model and statistical model. The structural model was simulated with one- and two-compartment models. As for statistical models, interindividual variability was described by an exponential function, while residual variability was described by additive, proportional, and additive plus proportional models.

#### 2.4.2 Covariate model

Following the establishment of the base model, a covariate analysis was undertaken. Scatter plots were employed to scrutinize the relationships between potential covariates and empirical Bayes estimates derived from the base model. To mitigate collinearity, variables exhibiting high correlation coefficients (r > 0.5) were not concurrently incorporated into the final model. Covariate screening was evaluated using a stepwise covariate modeling approach which includes forward inclusion and backward elimination steps. Population median values were employed for normalizing continuous covariates. A covariate was considered significant if the objective function value (OFV, defined as −2 times the log likelihood) decreased over 3.84 (*p* < 0.05) in forward inclusion and increased over 10.83 (*p* < 0.001) in backward elimination. The final model underwent refinement, incorporating additional criteria to ensure covariate integrity, including: 1) minimization of the objective function value (OFV), 2) enhancement of goodness-of-fit (GOF), 3) reduction of inter-individual variability resulting from covariate inclusion, and 4) confirmation of the clinical plausibility of the covariates. Categorical factors were accommodated using a piecewise model, while continuous factors underwent analysis employing linear, exponential, and power models.

$$P=\left\{\begin{array}{ll} \theta_{1}, & ifCOV=0\\ \theta_{1}\times \left(1+\theta_{2}\right), & ifCOV=1 \end{array}\right.$$

$$P=\theta_{1}+\theta_{2}\times \left(COV-{COV}_{median}\right)$$

$$P=\theta_{1}\times \mathrm{EXP}\left(\theta_{2}\times \left(COV-{COV}_{median}\right)\right)$$

$$P=\theta_{1}+{\left(\frac{COV}{{COV}_{median}}\right)}^{\theta_{2}}$$

#### 2.4.3 Model evaluation

The final model’s adequacy was appraised through a Goodness-of-fit (GOF) analysis, plotting observed concentration (DV) against both population-predicted concentration (PRED) and individual-predicted concentration (IPRED), along with conditional weighted residuals (CWRES) against PRED and time since the last dose. Furthermore, a nonparametric bootstrap analysis, which involved generating 1,000 random resamples from the original dataset, was conducted to evaluate the stability and reliability of the final model. The average parameter values obtained from the bootstrap analysis were then contrasted with the final estimates. Furthermore, a Prediction-corrected visual predictive check (pcVPC) visually evaluated the predictive performance. This involved simulating concentration profiles 10,000 times using the final model’s parameter estimates and plotting the 5th and 95th percentiles (90% prediction interval) against observed concentrations.

#### 2.4.4 Monte Carlo simulations of dosage regimens

The Monte Carlo simulations (MCS), consisting of 10,000 replicates, were performed using Crystal Ball 11.1.2.4 (Oracle, Redwood Shores, CA, United States), based on the parameter estimates derived from the final model. A VRC C_min_ of at least 2 mg·L^−1^for critically ill patients was recommended by the British Society for Medical Mycology [11]. To prevent adverse effects, a trough level ≤5 mg·L^−1^ is generally recommended. The probability of VRC target C_min_ attainment for different dosage regimens stratified by CRRT was estimated base on different loading and maintain dose.

## 3 Results

### 3.1 Patient demographics

A cohort comprising 72 individuals (54 males and 18 females) was enrolled, presenting a median age of 77. The demographic and clinical data of the participants are summarized in Table 1. Out of the 72 patients, 25 underwent continuous veno-venous hemofiltration (CVVH) therapy with blood flow rates between 150 and 180 mL·min-1 and dialysate flow set at 2 L·h^−1^. With regards to voriconazole (VRC) administration, 63 cases were evaluated, of which only 9 were for prophylactic purposes, and a total of 50 patients received an initial loading dose as part of their treatment plan. The predominant infecting pathogens identified were *Aspergillus spp*. (including *A. fumigatus, A. flavus, and Aspergillus niger*) as well as *Candida spp*. The dosages of VRC administered varied between 100 and 450 mg, given either once or twice daily. Most patients had two plasma concentration measurements of VRC and completed a 9-day treatment duration, whereas 33 patients had a single therapeutic drug monitoring (TDM) result. It is noteworthy that there was considerable variability in trough concentration levels among the patients, ranging from 0.15 to 11.0 mg L^−1^. The CYP2C19 phenotypes consisted of 39 IMs, 28 EMs, 4 PMs, and 1 RM, were divided into three groups (UM/EM, IM and PM) for PPK modeling. CYP3A4 genotypes were divided into group 1 (G/G), and group 2 (G/A and A/A). A Chi-square test and Hardy-Weinberg equilibrium analysis were conducted to evaluate the genotyping results of the four SNPs, with all SNPs adhering to Hardy-Weinberg equilibrium (p > 0.05) except for rs4244285 (p < 0.05), as detailed in Supplementary Tables S2.

**TABLE 1:** Demographics and clinical characteristics.

Characteristics	Modeling group (n = 72)
Gender (male/female), n	54/18
Age, year, (Median [IQR])	77 (69, 84)
Weight, kg, (Median [IQR])	66.5 (60, 70)
DV, mg/L, (Median [IQR])	3.6 (2.5, 5)
INR, (Median [IQR])	1.05 (0.98, 1.2)
ALT, IU/L, (Median [IQR])	21.4 (12.1, 35.36)
AST, IU/L, (Median [IQR])	26.78 (19.2, 43.68)
ALP, IU/L, (Median [IQR])	77.95 (57.6, 117.94)
GGT, IU/L, (Median [IQR])	65.53 (41.79, 136.32)
TBIL, μmol/L, (Median [IQR])	9.68 (6.35, 16.8)
DBIL, μmol/L, (Median [IQR])	3.75 (1.86, 8.98)
TP, g/L, (Median [IQR])	55 (49.82, 60.04)
ALB, g/L, (Median [IQR])	32.6 (30.61, 35.03)
GLO, g/L, (Median [IQR])	21.82 (17.89, 27.4)
UREA, mmol/L, (Median [IQR])	10.92 (7.38, 17.8)
SCR, μmol/L, (Median [IQR])	84 (56.08, 120.04)
CRP, mg/L, (Median [IQR])	60.4 (19.5, 108.5)
WBC, 10^9^/L, (Median [IQR])	9.6 (6.34, 14.24)
NEUT, 10^9^/L, (Median [IQR])	8.2 (4.91, 12.82)
HGB, g/L, (Median [IQR])	88 (76.5, 109)
HCT, %, (Median [IQR])	27.65 (24.1, 34.1)
PLT, 10^9^/L, (Median [IQR])	126 (73.63, 196)
GLU, mmol/L, (Median [IQR])	7.83 (5.75, 10)
ApoA1, g/L, (Median [IQR])	0.65 (0.49, 0.77)
ApoB, g/L, (Median [IQR])	0.7 (0.48, 0.85)
eGFR, ml·min^-1^/1.73 m^2^, (Median [IQR])	78.2 (48.39, 125.6)
CLcr, mL/min, (Median [IQR])	54.97 (36.04, 96.6)
CYP2C19 phenotypes, n (%) of patients	
Rapid metabolizer (RM)	1 (1.4)
Extensive metabolizer (EM)	28 (38.9)
Intermediate metabolizer (IM)	39 (54.2)
Poor metabolizer (PM)	4 (5.6)
CYP3A4 rs4646437 genotypes, n (%) of patients	
G/G	59 (81.9)
G/A	12 (16.7)
A/A	1 (1.4)
CRRT during voriconazole therapy, n (%) of patients	
CRRT	25 (34.7)
CAPA diagnosis, n (%) of patients	
Probable	63 (87.5)
Possible	9 (12.5)
Underlying disease, n (%) of patients	
Hypoproteinemia	45 (62.5)
Diabetes mellitus	36 (50)
Hypertension	44 (61.1)
Respiratory failure	44 (61.1)
Concomitant medication, n (%) of patients	
Paxlovid	12 (16.7)
Azvudine	8 (11.1)
Clopidogrel	6 (8.3)
PPIs	58 (80.6)
Omeprazole	33 (45.8)
Esomeprazole	7 (9.7)
Lansoprazole	12 (16.7)
Pantoprazole	6 (8.3)
CCBs	21 (29.2)
Amlodipine	7 (9.7)
Nifedipine	3 (4.2)
Nicardipine	11 (15.2)
Glucocorticoids	61 (84.7)
Methylprednisolone	27 (37.5)
Prednisolone	6 (8.3)
Dexamethasone	17 (23.6)
Hydrocortisone	11 (15.2)
Voriconazole trough concentration, n (%) of concentration points (n = 150)	
<2 mmol/L	23 (15.3)
2∼5 mmol/L	92 (61.3)
>5 mmol/L	35 (23.3)
Outcomes, n (%) of patients	
Expired	33 (45.8)
Discharged living	25 (34.7)
Discharge against medical advice	14 (19.4)

^*^Values are median (IQR) or n (%). ALB, albumin; ALP, alkaline phosphatase; ALT, alanine aminotransferase1; APOA1, apolipoprotein A; APOB, apolipoprotein B; AST, aspartate aminotransferase; CCBs, calcium channel blockers; CREA, creatinine; CRP, C-reactive protein; CRRT, continuous renal replacement therapy; CLCR, creatinine clearance rate (according to Cockcroft–Gault formulation); DBIL, direct bilirubin; DV, the observed value; GFR, glomerular filtration rate; GGT, gamma-glutamyl transpeptidase; GLO, globulin; GLU, blood glucose; HGB, hemoglobin; HCT, hematocrit; INR, international normalized ratio; SCR, serum creatinine; TBIL, total bilirubin; TP, total protein; NEUT, neutrophil count; PLT, platelet; PPIs, proton pump inhibitors; WBC, white blood cell count.

### 3.2 Population pharmacokinetic modeling

One-compartment pharmacokinetic model with first-order elimination could adequately describe the concentration-time data and the residual error was best characterized by an addictive error model. The major PK parameters included clearance (CL) and volume of distribution (V). In our basic model, the shrinkage of V was high (58%), which means the V parameter was not significant estimate inter individual variability. Hence, we fixed the inter individual variability of V as zero. Forward and backward covariate selection processes were shown in Supplementary Table S3. In general, the final population PK model and bootstrap results were shown in Table 2. The bootstrap results demonstrate a high level of reliability in the final model, with 991 out of 1,000 bootstrap trials yielding successful outcomes. It is worth noting that all parameter estimates align with the standard values of the definitive model, and that the 95% confidence interval perfectly coincides with the parameters of the final model. The equations representing the final model are provided below:

$$CL\left(L/h\right)=\left\{\begin{array}{l} 3.17*e^{0.0768}*{\left(\frac{PLT}{121}\right)}^{0.248}*{\left(\frac{CRP}{55.23}\right)}^{-0.183}*{\left(\frac{GGT}{68.72}\right)}^{0.2928}*{\left(\frac{AST}{29.91}\right)}^{-0.227}\;,CRRT=0\\ 3.17*e^{0.0768}*{\left(\frac{PLT}{121}\right)}^{0.248}*{\left(\frac{CRP}{55.23}\right)}^{-0.183}*{\left(\frac{GGT}{68.72}\right)}^{0.2928}*{\left(\frac{AST}{29.91}\right)}^{-0.227}*CRRT*1.617,CRRT=1 \end{array}\right.$$

$$V\left(L\right)=135$$

**TABLE 2:** Population pharmacokinetics parameter estimates of the base and final model with bootstrapping data on voriconazole.

Parameters	Base model		Final model		Bootstrap	
	Estimate	RSE (%)	Estimate	RSE (%)	Median	95% CI
CL	3.67	6.6	3.17	6.1	3.176	2.76–3.57
V	140	26.4	135	22.1	130.919	54.37–200.50
θ_3_ (PLT on CL)	NA	NA	0.248	32.7	0.235	0.043–0.404
θ_4_ (CRP on CL)	NA	NA	−0.183	18.2	−0.181	−0.258 ∼ (−0.113)
θ_5_ (GGT on CL)	NA	NA	0.292	20.4	0.288	0.177–0.442
θ_6_ (CRRT on CL)	NA	NA	0.617	31.9	0.57	0.232–1.105
θ_7_ (AST on CL)	NA	NA	−0.227	34.2	−0.224	−0.419 ∼ (−0.078)
Interindividual variability						
ω^2^_*CL*_	0.212	29.4	0.0768	34.4	0.0678	0.018–0.126
ω^2^_*V*_	0 FIX	NA	0 FIX	NA	0 FIX	NA
Residual variability						
Additive error	2.3	10.2	1.87	16.4	1.771	1.212–2.444

^*^CL, apparent clearance; V, apparent volume of distribution; PLT, platelet (10^9^/L); CRP, C-reactive protein (mg/L); GGT, glutamyl transpeptidase (IU/L); CRRT, 1, continuous renal replacement therapy was taken, and CRRT, 0, continuous renal replacement therapy was not taken; AST, is aspartate aminotransferase (IU/L); ω^2^_CL_, ω^2^_V_ are variance estimates of the interindividual variability of CL, and V, respectively; Additive error is the variance estimate of the variance of the summed residual variance; and RSE, is the standard error of the residual variance.

**FIGURE 1:**
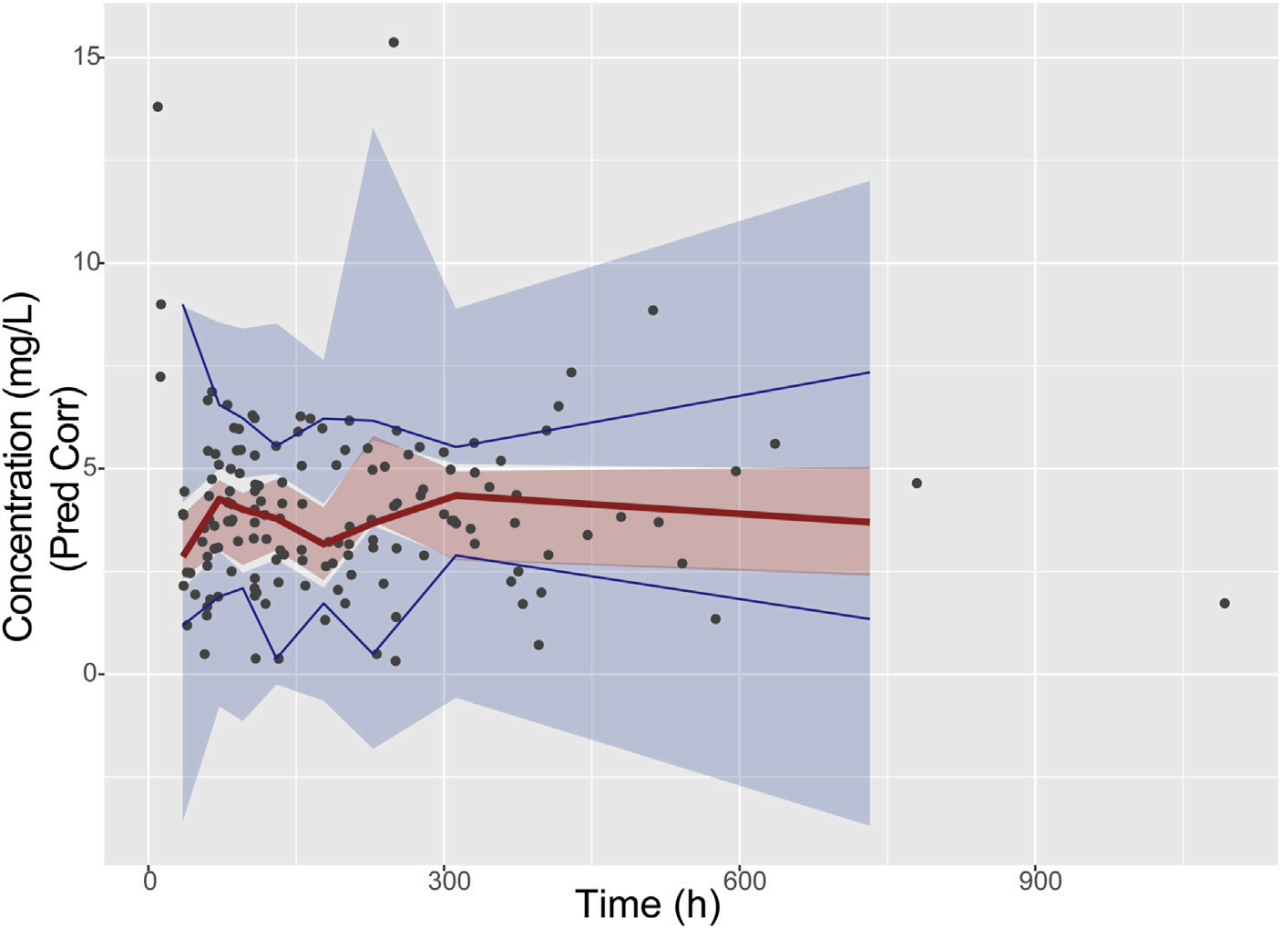
Prediction-corrected VPC (pcVPC) of the final model. The black solid points represent the dependent variable; upper, middle, and lower solid line represent the 95th, 50th and fifth percentiles of the observed concentrations, respectively; The upper, middle, and lower shaded sections represent the 95% confidence interval for the 95th, 50th, and fifth percentiles of the simulated concentrations, respectively.

The VPC result in Figure 1 demonstrate that the observed median (represented by a solid line) and the observed 5th and 95th percentiles (indicated by dashed lines) are reasonably situated within the simulation-based prediction intervals (depicted as shaded areas). All of the bootstrap, GOF and VPC results indicated the final model had good stability and robustness.

**FIGURE 2:**
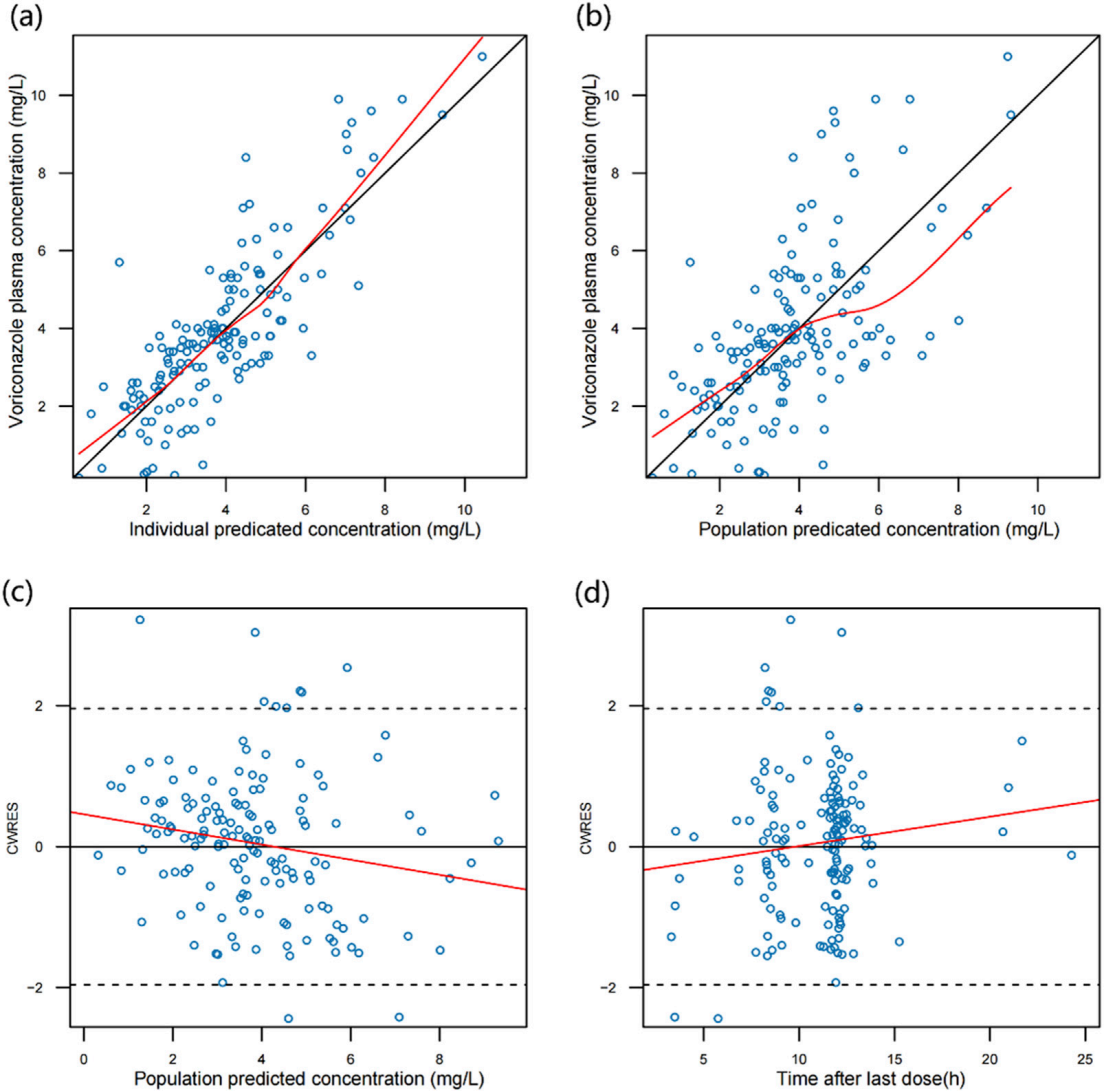
Diagnostic goodness-of-fit plots for the final model. **(A)** Scatter plot of voriconazole plasma concentration and individual predicted values. **(B)** Scatter plot of voriconazole plasma concentration and population predicted values. **(C)** Scatter plot of conditional weighted residuals and population predicted values. **(D)** Scatter plot of conditional weighted residuals and time from last dose. The black solid and dashed line are the reference line, and the red solid line is the LOWESS trend line.

The goodness-of fit plots of final model were shown in Figure 2. PRED and IPRED had good correlations with DV. The scatter points in the PRED and IPRED plots are more evenly scattered around the reference line (y = x); as seen in the CWRES plots of PRED and TAD, the scatter points are most evenly distributed within ±2, and the LOWESS regression line is close to y = 0.

### 3.3 Monte Carlo simulations of dosage regimens

The likelihood of achieving the VRC target C_min_ with different loading and maintenance dosing regimens, stratified by CRRT, is presented in Table 3, 4. The results suggest that all dosing schedules administered intravenously twice daily were sufficient to meet the therapeutic needs for non-CRRT patients. Conversely, for those undergoing CRRT, it is essential to administer a minimum of 5 mg/kg intravenously twice daily to achieve the VRC therapeutic range, ensuring that patients reach the minimum target concentration of 2 mg L^−1^.

**TABLE 3:** Probability of the *C*_24_ attaining the therapeutic range (2–5 mg/L) with different intravenous voriconazole loading doses based on body weight in CRRT and non-CRRT group.

Loading dose (for the first 24 h)	Probability (%)					
	CRRT			Non-CRRT		
	<2 mg/L	2–5 mg/L	>5 mg/L	<2 mg/L	2–5 mg/L	>5 mg/L
6 mg/kg q12 h	0.18	99.64	0.18	0	99.42	0.58
5 mg/kg q12 h	1.49	98.51	0	0.18	99.82	0
4 mg/kg q12 h	16.88	83.12	0	4.16	95.84	0

*C*_24_: voriconazole plasma concentrations at 24 h, namely, trough concentrations (*C*_min_).

**TABLE 4:** Probability of the *C*_*trough*_ *,*_ss_ attaining the therapeutic range (2–5 mg/L) with different intravenous voriconazole maintain doses based on body weight in CRRT and non-CRRT group.

Maintenance dose	Probability (%)					
	CRRT			Non-CRRT		
	<2 mg/L	2–5 mg/L	>5 mg/L	<2 mg/L	2–5 mg/L	>5 mg/L
6 mg/kg q12 h	0.02	53.27	46.71	0	0.8	99.2
5 mg/kg q12 h	0.24	77.24	22.52	0	5.98	94.02
4 mg/kg q12 h	2.79	90.48	6.73	0	28.03	71.97
3 mg/kg q12 h	21.75	77.29	0.96	0.04	71.71	28.26
2 mg/kg q12 h	78.28	21.66	0.05	5.92	91.33	2.75

*C*_*trough*_ *,*_ss_: voriconazole plasma concentrations at steady state, namely, trough concentrations (*C*_*trough*_ *,*_ss_).

Regarding the maintenance dosage, 2 mg/kg administered intravenously twice daily was sufficient for patients not undergoing CRRT, whereas a higher dosing regimen of 4 mg/kg intravenously twice daily is necessary for CRRT patients to achieve the therapeutic range for VRC effectiveness.

## 4 Discussion

The prevalent transmissibility of new coronaviruses and the elevated mortality rate associated with CAPA have garnered significant global interest over the last 5 years. This study represents the inaugural development of a Population Pharmacokinetic (PPK) model for voriconazole within a cohort of CAPA patients. It aims to identify and quantify the determinants influencing the pharmacokinetic properties of voriconazole, thereby offering a dependable reference for dosage modulation in clinical practices involving voriconazole.

It was found that one-compartment model with first-order elimination best characterized PK of intravenous VRC, which is consistent with the results of several studies, including (Wang et al., 2024) and Simon (Simon et al., 2021). The standard values observed for the CL and V parameters in this investigation were 3.17 L/h and 135 L, respectively, which correspond to the previously reported ranges of 2.88–4.28 L/h and 93.4–200 L in other studies ^[19–22]^. This study also explored the effect of other covariates on VRC PK in patients with CAPA and found that C-reactive protein (CRP), glutamyl transpeptidase (GGT), platelet count (PLT) and aspartate aminotransferase (AST), as well as the use of continuous renal replacement therapy (CRRT), had a significant effect on CL.

Unexpectedly, we observed an increase in voriconazole clearance (CL) when continuous renal replacement therapy (CRRT) was employed, a finding that is partially supported by the insights of Wang and Herve. The average CL of voriconazole under the influence of CRRT is approximately 5.13 L/h, which markedly surpasses the levels recorded in patients not undergoing CRRT. We share Wang’s perspective that the previous researches present varying conclusions regarding the role of CRRT in voriconazole pharmacokinetics, likely attributable to the limited sample sizes in these investigations, which hinder the establishment of a consistent and reliable outcome. Wang’s analysis encompassed 104 individuals, yielding 186 concentration measurements, which indicated an enhancement in voriconazole CL associated with CRRT. Although the cohort of patients receiving CRRT in our study was smaller than Wang’s, but still representing 34.7% of our total participants, thereby underscoring the substantial influence of CRRT on the clearance of voriconazole.

VRC possesses a relatively low molecular weight of 349 Da, exhibits moderate lipophilicity, has a low plasma protein binding ratio of 58%, and demonstrates a high volume of distribution of 4.6 L/kg. A total of 25 patients with CRRT were included in the study, and all CRRTs were performed in the CVVH mode, a mode in which solute convection passes through membranes with a pressure gradient, and drugs with higher molecular weights can also be effectively cleared by hemofiltration. In addition, the hypoalbuminemia of the study population, the variable physio-pathological conditions of critically ill patients, and the lower distribution volume estimated by the model than that of the healthy population make the filtration of VRC more difficult to predict. Quintard (Quintard et al., 2008) found the high-volume CVVH patterns affect VRC elimination, multi-organ failure leading to saturation of VRC metabolic clearance, small amounts of VRC may be adsorbed to the hemofiltration membrane (Kiser et al., 2015). Thus, determining whether voriconazole is adsorbed onto the hemofilter membrane in the same way as onto the ECMO membrane may be important for determining the increase in voriconazole clearance caused by CRRT.

CRP significantly affects CL similar to several studies (Pepys and Hirschfield, 2003; Encalada Ventura et al., 2015; Jiang et al., 2022; Boglione-Kerrien et al., 2023), CRP is an acute protein in the plasma in the presence of infection or tissue damage to the organism, a non-specific inflammatory marker produced mainly by hepatocytes, especially in hepatocyte injury, infection and inflammation increase dramatically, plasma CRP levels can reflect the severity of inflammation. Van Den Born et al. (2023) performed PPK modeling using 1060 VRC blood concentration values from 54 patients and screened the only covariate which was CRP, based on the model it can be concluded that for every increase of CRP by 150 mg/L, the CL of VRC was reduced by 50%. Encalada Ventura et al. (2015) defined CRP ≥100 mg/L as a factor that severely affects the metabolism of VRC. VRC metabolism, with VRC blood levels increasing by 0.021 mg/L and VRC metabolism decreasing by 1% with increasing unit CRP levels. Compared to studies that included CYP2C19 genotype as a covariate, the patient population in this study consisted of 45 (62.5%) hypoalbuminemia patients, which is similar to the findings of Li et al. (2024), in which CRP significantly affects the steady-state concentration of VRC more than genotype in patients with hypoalbuminemia. Xu’s study (Hao et al., 2023) demonstrated that, in the moderately to severely infected Chinese with a CRP ≥40 mg/L population, CRP was considered to be an independent factor affecting the steady-state concentration of VRC, thus masking the CYP2C19 gene polymorphism that significantly affected the VRC concentration in patients with lower indicators of infection. The median (IQR) of CRP in this study was 84 (56.08, 120.04) mg/L, mostly in patients with severe infections, and cytokines in the inflammatory state inhibit the expression of the CYP enzyme levels and activities (Simon et al., 2021), and CRP affects CL more significantly than genotype.

In the current research, a positive association was identified between platelet count and voriconazole clearance, aligning with the findings of Tang et al. (2021). It has been documented that platelet count is linked to liver function. As liver function deteriorates, the resultant portal hypertension and reduced thrombopoietin levels contribute to a decline in platelet count. Consequently, the platelet count observed in our study may merely reflect the state of liver function. Predictably, AST and GGT, which serve as widely recognized biomarkers of liver function, were integrated into the model, a fact that has been substantiated by several prior studies (Li et al., 2017; Chantharit et al., 2020).

The metabolic enzymes analyzed in the prior study encompassed CYP2C19 and CYP3A4. Additionally, the concomitantly administered medications that exhibited drug interactions comprised glucocorticoids, proton pump inhibitors, calcium channel blockers, clopidogrel, the antiviral agent paxlovid, the immunosuppressant tacrolimus, and cyclosporine. Voriconazole, functioning as a hepatic enzyme and P-glycoprotein inhibitor, was found to increase the plasma concentrations of concomitantly administered medications, as evidenced by tacrolimus and omeprazole. Therefore, data on drug interactions was thoroughly collected initially, but the number of drug combinations was limited. Ultimately, only glucocorticoids and proton pump inhibitors were included, with few combinations in each category, which could not be integrated into the model after broad categorization.

This research presents certain limitations that warrant further investigation and refinement. 1) The study is confined to a single center with a brief duration, and the diagnosis of patients with COVID-19-associated pulmonary aspergillosis is multidimensional. The sample size suitable for exclusions is constrained, precluding external validation of the model; hence, multi-center and more heterogeneous patient cohorts are necessary to confirm the model’s applicability. 2) A majority of the participants were critically ill individuals who had been bedbound for an extended period, resulting in challenges in obtaining accurate weight data, with over 30% missing values. Consequently, weight was not incorporated into the modeling process, leading to sparse sampling (1–2 blood collection points) among patients. This resulted in a larger inter-individual variability contraction value for volume distribution (V), and some data in basic and covariate models were omitted, suggesting that the findings may not accurately represent the pharmacokinetic characteristics of this cohort. 3) The study did not identify significant genotypic effects on the pharmacokinetic parameters of voriconazole (VRC), which may be attributable to a limited number of patients classified as poor metabolizers (PM) and ultra-rapid metabolizers (UM), thus hindering the ability to draw definitive conclusions.

## 5 Conclusion

In this study, the PPK model of VRC in CAPA patients was established for the first time, and the *in vivo* process of VRC could be described by one-compartment model with first-order elimination, and five covariates that significantly affect VRC metabolism, such as CRRT, CRP, GGT, PLT, and AST. The final internal validation results of the model are good, indicating that the model is stable and has good predictive performance. It helps to individualize and rationalize the use of voriconazole in CAPA patients, especially informing the treatment of patients receiving CRRT.

## Data Availability

The original contributions presented in the study are included in the article/Supplementary Material, further inquiries can be directed to the corresponding author.
