# Supplementary material for: Population pharmacokinetics and dose optimization of voriconazole in patients with COVID-19-associated pulmonary aspergillosis
Source: Front Pharmacol. 2025 Apr 9;16:1554370. doi: 10.3389/fphar.2025.1554370 (PMC12014539; doi:10.3389/fphar.2025.1554370)
Supplement: Supplementary file 1 [file Table1.docx]

Electronic Supplementary Material

of

Population Pharmacokinetics and Dose Optimization of Voriconazole in Patients with COVID-19-Associated Pulmonary Aspergillosis

Hui Wang ^1#^, Yue Shen^3#^, Xuemei Luo^2^, Lu Jin^2^, Huaijun Zhu^2^*, Jing Wang^2^*

^1^ Department of Infection Management, The Affiliated Nanjing Drum Tower Hospital of Nanjing University Medical School, 321 Zhongshan Road, Nanjing, Jiangsu 210008, China

^2^ Department of Pharmacy, The Affiliated Nanjing Drum Tower Hospital of Nanjing University Medical School, 321 Zhongshan Road, Nanjing, Jiangsu 210008, China

^3^ Department of Pharmacy, The First Affiliated Hospital of Xi'an Jiaotong University, Xi'an, Shaanxi, China.

#These authors contributed to the work equally and should be regarded as co-first authors.

*Corresponding Author E-mail:

huaijun.zhu@gmail.com (Huaijun Zhu);

18351975003@163.com (Jing Wang);

**Table S1.** Primers used for SNP genotyping

| Genes | Locus | rs no. | Primers |
| --- | --- | --- | --- |
| *CYP2C19* | *2 | rs4244285 | F 5’-AAAATTTCCCCATCAAGATATAC-3’  R 5’-TCAGGAAGCAATCAATAAAGTCC-3’ |
|  | *3 | rs4986893 | F 5’-CTTTCATCCTGGGCTGTGCT-3’  R 5’-CCCCATGGCTGTCTAGGCA-3’ |
|  | *17 | rs12248560 | F 5’-TTTTATGAACAGGATGAATGTGG-3’  R 5’-CAAATGGGAAAAGGGAGACC-3’ |
| *CYP3A4* | c.671-202C>T | rs4646437 | F 5’-TGTGAATGTGCAAAATTTACCTG-3’  R 5’-TGATTTGGGTTATTCTAGGAGAC-3’ |

**Table S2.** Hardy-Weinberg Equilibrium analysis for SNP genotyping

| Gene | SNPs | Genotype frequency, n (%) | | HWE-*p* |
| --- | --- | --- | --- | --- |
| *CYP2C19* | rs4244285 | GG | 31(0.43) | 0.003 |
|  |  | AG | 41(0.57) |  |
|  | rs4986893 | AG | 6(0.08) | 0.934 |
|  |  | GG | 66(0.92) |  |
|  | rs12248560 | CC | 71(0.99) | 0.998 |
|  |  | TC | 1(0.01) |  |
| *CYP3A4* | rs4646437 | GG | 59(0.82) | 0.912 |
|  |  | AG | 12(0.17) |  |
|  |  | AA | 1(0.01) |  |

**Table S3.** Summary of forward inclusion and backward elimination during model building

| Model No. | | Model description | OFV | △OFV | | *P* Value | | Decision | |
| --- | --- | --- | --- | --- | --- | --- | --- | --- | --- |
| Forward inclusion | | |  | |  | |  | |  |
| 1 | Base model | | 357.968 | | / | | / | | / |
| 2 | Add PLT on CL in model 1 | | 342.294 | | 15.674 | | <0.001 | | Accept |
| 3 | Add CRP on CL in model 2 | | 333.224 | | 9.07 | | <0.01 | | Accept |
| 4 | Add GGT on CL in model 3 | | 319.093 | | 14.131 | | <0.001 | | Accept |
| 5 | Add CRRT on CL in model 4 | | 307.909 | | 11.184 | | <0.001 | | Accept |
| 6 | Add AST on CL in model 5 | | 294.231 | | 13.678 | | <0.001 | | Accept |
| 7 | Add eGFR on CL in model 6 | | 285.656 | | 8.575 | | <0.01 | | Accept |
| 8 | Add TP on CL in model 7 | | 279.821 | | 5.835 | | <0.05 | | Accept |
| Backward elimination | | |  | |  |  | | |  |
| 9 | Remove TP on CL from model 8 | | 285.656 | | 5.835 | | >0.001 | | Accept |
| 10 | Remove eGFR on CL from model 9 | | 294.231 | | 8.575 | | >0.001 | | Accept |
| 11 | Remove AST on CL from model 10 | | 307.909 | | 13.679 | | <0.001 | | Reject |
| 12 | Remove CRP on CL from model 10 | | 316.952 | | 22.721 | | <0.001 | | Reject |
| 13 | Remove CRRT on CL from model 10 | | 309.956 | | 15.725 | | <0.001 | | Reject |
| 14 | Remove GGT on CL from model 10 | | 317.334 | | 23.103 | | <0.001 | | Reject |
| 15 | Remove PLT on CL from model 10 | | 311.818 | | 17.587 | | <0.001 | | Reject |
